# Designing Consent Processes for Research With Older Adults Who May Lack Decisional Capacity: A Guide for Investigators

**DOI:** 10.1111/jgs.70473

**Published:** 2026-05-07

**Authors:** Emily A. Largent, Stephanie R. Morain, Jason Karlawish

**Affiliations:** ^1^ Department of Medical Ethics and Health Policy University of Pennsylvania Perelman School of Medicine Philadelphia Pennsylvania USA; ^2^ Berman Institute of Bioethics Johns Hopkins University Baltimore Maryland USA; ^3^ Department of Medicine, Department of Medical Ethics and Health Policy, Department of Neurology University of Pennsylvania Perelman School of Medicine Philadelphia Pennsylvania USA

**Keywords:** capacity, informed consent, research ethics

## Abstract

Securing a participant's voluntary, informed consent is widely acknowledged as a requirement of ethical research. Yet, challenges can arise, particularly when a researcher seeks to enroll older adults who may lack the capacity to consent to research participation due to cognitive impairment. To address this challenge, this article outlines a nine‐step framework for designing consent processes: (1) identify the participants in the study and assess whether some or all might reasonably be expected to lack decisional capacity; (2) determine whether enrolling participants who lack decisional capacity is justifiable; (3) determine whether informed consent is needed for some or all research procedures; if consent is required, (4) prepare the information that will be provided to prospective participants; (5) make both the information and the consent process accessible; (6) specify how prospective participants' decisional capacity will be assessed; (7) specify how surrogates will be identified and contacted when prospective participants are determined to lack decisional capacity; (8) determine how to involve prospective participants who lack decisional capacity in the decision‐making process, such as through assent or dissent; and as appropriate, (9) consider how capacity and consent might change across the duration of the study. This framework can help researchers anticipate and address common challenges and ensure they fulfill ethical and regulatory obligations. Applications of this framework include writing the human subjects portion of grant applications, preparing protocols for submission to an Institutional Review Board, and informing research practice.

Consent is generally a requirement for the ethical conduct of human subjects research [1]. Illustrating this, the National Commission for the Protection of Human Subjects of Biomedical and Behavioral Research was tasked with identifying basic ethical principles governing research; the resulting *Belmont Report*—which undergirds federal human subjects research regulations in the United States—explains that the principle of “[r]espect for persons requires that subjects, to the degree that they are capable, be given the opportunity to choose what shall or shall not happen to them.” [2] The purpose of the consent process is to give prospective participants the opportunity to make an informed, voluntary decision that participation in research is or is not consistent with their values and interests [3].

From the researcher's perspective, the consent process has three elements: (1) disclosure of relevant information by the researcher (or researcher's designee), (2) facilitation of understanding of what has been disclosed, and (3) promotion of a voluntary decision to participate (or not). Regarding the first of these, research regulations and guidance documents outline the information that must be provided to prospective participants. The latter two elements—understanding and voluntariness—reflect the cognitive and volitional dimensions of consent, respectively. Decisional capacity is a threshold requirement for the consent process, as prospective participants should be capable of making a truly informed choice, and their decision should be made free of coercion or undue influence.

While this is straightforward in theory, researchers seeking consent from prospective participants often encounter challenges in practice. A clear instance of this is when researchers seek to enroll older adults who are likely or known to be cognitively impaired in their studies [4]. Cognition is not the same as decision‐making capacity, though the two are correlated. Multiple studies measuring decisional capacity show that as cognition declines, so too does performance on measures of capacity. When some or all prospective research participants may lack the capacity to consent to research participation, researchers need a clear plan.

To help researchers accomplish this, we provide a brief primer on decisional capacity and then present a nine‐step framework to guide the design of consent processes when decisional capacity is or may be at issue. This article assumes the research is governed by the U.S. Federal Policy for the Protection of Human Subjects or “Common Rule,” though many of the points apply generally or could be adapted to different regulatory frameworks protecting research participants.

## 1 Decisional Capacity

Capacity is generally understood to encompass four relevant decisional abilities: comprehension, appreciation, reasoning, and choice [4]. Comprehension is the ability to grasp the fundamental meaning of relevant information. By contrast, appreciation is the ability to acknowledge information is relevant to *oneself*. To illustrate the difference, consider the following example. A person who understands randomization might liken it to “flipping of a coin,” while a person who appreciates randomization might state, “I could receive the study drug, or I could receive the placebo. It's totally random.” Reasoning is the ability to rationally weigh options, whether comparatively (*X* vs. *Y*) or consequentially (if *A*, then *B*). Finally, choice refers to the ability to consistently communicate a choice.

Setting aside legal processes such as guardianship in which *global* determinations of competency may be made by a judge, decisional capacity is understood to be task and context specific. When decisions are associated with higher risks, individuals may be held to a higher standard than when decisions are associated with lower risks [5]. In practice, this means that an individual may have capacity to make some research‐related decisions (e.g., whether to enroll in a one‐time interview study about their experience in an adult day program) while simultaneously lacking capacity to make others (e.g., whether to enroll in a randomized controlled trial [RCT] of an investigational new drug for Alzheimer's). Thus, researchers must specify “the capacity to do *X*,” where *X* is the decision at hand. In other words, one should never end a sentence with capacity—as in “She lacks capacity.” Instead, one should address what must be decided—as in “She lacks capacity to consent to this RCT.” Lest it seem we are failing to heed our own advice, when we speak of “capacity” or “decisional capacity” in this article, it should be understood as “capacity to consent to participation in the researcher's particular study.”

Individuals with capacity are empowered to make their own decisions. By contrast, individuals who lack capacity need others to decide for them. Some individuals fall into an intermediate category, that of “marginal capacity,” and may struggle to make decisions independently [6]. Marginal capacity is an ethically and practically important designation, as accommodations can improve the quality of individuals decision making and enable them to make their own decisions [7]. For instance, a consent process employing memory and organizational aids can improve the ability of persons with mild stage dementia to consent to an early phase Alzheimer's clinical trial [8]. Having another person support decision‐making processes can also allow an individual with marginal capacity to make their own decision about research participation [7].

Before proceeding, we offer some additional guidance. Because cognition is correlated with but not the same as decisional capacity, neither a diagnosis of cognitive impairment (e.g., mild cognitive impairment [MCI] or dementia) nor diagnosis with a condition that causes cognitive impairment (e.g., Alzheimer's or Parkinson's) necessarily means that an individual lacks decisional capacity. Though incapacity is common, many people with dementia can make their own research‐related decisions [9]. Similarly, cognitive test scores—for example, from the Mini‐Mental State Examination (MMSE) or the Montreal Cognitive Assessment (MoCA)—generally should not be used to establish that an individual has or lacks decisional capacity, though they may establish pre‐test probability.

## 2 Framework for Developing a Study‐Specific Consent Process

Here, we outline nine steps a researcher or research team should take when developing a consent process (Box 1). While designed for researchers intending to enroll individuals lacking decisional capacity in their studies, many steps are also relevant to researchers intending to exclude such individuals (e.g., determining whether exclusion is justified [STEP 2] and, if so, having a plan to assess capacity in order to determine whom to exclude [STEP 6]).

BOX 1 **Framework for designing consent processes for research that enrolls older adults who may lack decisional capacity.**

1. Identify the study participants and assess the likelihood some or all may lack decisional capacity.
2. Determine whether enrolling participants lacking decisional capacity is justifiable.
3. Determine whether informed consent is needed for some or all research procedures.
4. Prepare the information that will be provided to prospective participants.
5. Make the information provided to prospective participants and the consent process accessible.
6. Specify the process for assessing prospective participants' decisional capacity.
7. Specify how surrogates will be identified and contacted when prospective participants are determined to lack decisional capacity.
8. Determine whether there are ways to involve prospective participants who lack decisional capacity in the decision‐making process.
9. In longitudinal studies, consider how capacity and consent might change over time.

### 2.1 STEP 1: Identify the Study Participants and Assess the Likelihood Some or All May Lack Decisional Capacity

The first step in designing a consent process is determining *who* needs to give informed consent. Stated simply, whose data will be obtained through intervention or interaction or whose identifiable data will be used? Consider a researcher who has developed an educational module on diagnosing cognitive impairment for use by primary care providers (PCPs). If the researcher is asking whether PCPs who complete the educational module report greater self‐efficacy when diagnosing cognitive impairment compared to the PCPs who don't complete the module, the participants are the PCPs. If the researcher is also asking whether patients seen by PCPs who have completed the educational module are more likely to be diagnosed with cognitive impairment, then both the patients and the PCPs are research participants, as the researcher is collecting data about both groups.

The prevalence of cognitive impairment within the study population should inform the approach to capacity assessment. In some populations, such as people living with dementia, cognitive impairment is universal and so capacity needs to be universally assessed. In other populations, impairment is not universal or even common; in these cases, capacity does not need to be routinely assessed, though all adults should be asked basic questions to assure they understand key points. The presence of cognitive impairment—from any cause—should prompt an assessment of decisional capacity.

### 2.2 STEP 2: Determine Whether Enrolling Participants Lacking Decisional Capacity Is Justifiable

In research ethics, vulnerable populations are those with a feature that places them at greater risk of being wronged or harmed during research and so have a claim to additional or special protections [10]. Individuals lacking decisional capacity are vulnerable because they cannot make their own decisions about whether participation in research is consistent with their values and wishes. (Special protections for them include identifying someone, a surrogate, who can make decisions on their behalf [STEP7]).

Inclusion of those lacking decisional capacity in a study requires a sound scientific or ethical justification. Potential justifications—for example, that the research is relevant to them and could not be conducted with a less vulnerable population or that their inclusion promotes generalizability—are discussed in Box 2. Some reasons for enrolling individuals who lack decisional capacity *cannot* stand up to scrutiny; those lacking capacity should never, for instance, be enrolled in a study simply because it is convenient for the researcher.

BOX 2 **Potential justifications for enrolling individuals who lack decisional capacity.**

A strong justification for enrolling people who lack decisional capacity is that (1) the research is directly related to a condition or conditions affecting them, and also that (2) it is not possible to conduct this research with other, less vulnerable persons. For example, imagine a researcher wants to evaluate a behavioral intervention to reduce delirium in hospitalized older adults. Enrolling people lacking decisional capacity would be justified because it would be impossible to conduct this study without enrolling people with delirium, which adversely affects capacity, and the research has the potential to benefit them—or persons like them—in the future.

In some instances, condition (1) from the prior paragraph will be satisfied while condition (2) is not. When this occurs, it will generally be advisable to proceed by enrolling only those less vulnerable individuals who have decisional capacity. This may, for instance, be advisable if the study is evaluating a novel intervention, the risks and benefits of which are not yet well understood. In such cases, conducting a study with less vulnerable individuals will provide important preliminary data for future research with more vulnerable individuals.

Researchers might reasonably object that, although their research could be conducted by enrolling only individuals with decisional capacity, those with decisional capacity meaningfully differ from those who lack it. Stated otherwise, though condition (2) would not strictly speaking be satisfied, enrolling only individuals who retain decisional capacity would substantially limit the generalizability of the resulting data. In some cases, and depending on features of the study in question, threats to generalizability might justify enrolling both those who have and those who lack decisional capacity. Imagine, for example, that a researcher is proposing to study whether a U.S. Food and Drug Administration (FDA)‐approved drug can be used “off label” to reduce apathy in people living with Alzheimer's. If the researcher only enrolls people with decisional capacity—that is, less severe disease—the results might not be generalizable to people with more severe disease, who also experience a substantial burden of apathy. To promote scientific rigor and because (we will assume, given prior FDA approval) the safety of the drug is already well established, the researchers would likely be justified in enrolling individuals across the spectrum of disease.

Another possibility is that considerations of justice favor enrolling people who lack decisional capacity, even if their inclusion is not scientifically necessary. This could arise if participation in the research offers participants a prospect of direct benefit—that is, benefits to their health or well‐being—available only in the context of research. Imagine that a researcher is studying a promising vaccine for a condition like Group A *Streptococcus*, which is a serious cause of illness and death amongst older adults. We will assume for this example that there is no reason to think those who have and those who lack decisional capacity meaningfully differ from one another in ways that would affect the generalizability of an RCT; it cannot be argued that enrolling individuals lacking decisional capacity is required scientifically. Yet, given the burden of illness amongst all older adults and the prospect of direct benefit to participants, an appeal to justice may justify enrolling individuals lacking decisional capacity.

### 2.3 STEP 3: Determine Whether Informed Consent Is Needed for Some Or All Research Procedures

Next, it is essential to ask whether consent is needed for all research procedures. Typically, the answer to this question is a straightforward “yes,” and the researcher can proceed to STEP 4.

There may, however, be circumstances in which a waiver of informed consent is appropriate for some or all research procedures. For example, many pragmatic clinical trials are conducted with waivers of informed consent [11]. In such a case, the remaining steps of this framework will not apply to those research procedures covered by the waiver. Other ethical and regulatory considerations will, however, need to be addressed [11].

### 2.4 STEP 4: Prepare the Information That Will Be Provided to Prospective Participants

In the United States, the Common Rule outlines information that needs to be disclosed to prospective participants during the consent process [12]. This information must be presented, either orally or in writing, with sufficient details to facilitate prospective participants' understanding of why they might or might not want to participate. Because this information is necessarily study‐specific, we will not discuss it at greater length here. An Institutional Review Board (IRB) will review consent materials—and may request modifications—to ensure they satisfy the regulatory requirements.

### 2.5 STEP 5: Make Both the Information Provided to Prospective Participants and the Consent Process Accessible

Consent materials can be difficult for many prospective participants, even those with capacity, to understand. Therefore, the way the information from STEP 4 is conveyed is as important as the information itself. Best practices include providing information in plain language, avoiding use of jargon and acronyms, keeping written materials at (or a below) a 6th to 8th grade reading level, and allowing plenty of time to process information and ask questions [13].

If possible, researchers should allow—or even encourage—a family member or friend to be present with the prospective participant during the consent process. Empirical studies show that individuals with dementia and their surrogates decide whether to participate in research through a collaborative process, with surrogates playing an ever‐greater role as cognition worsens [14, 15, 16, 17]. Allowing a family member or friend to be present is, therefore, consistent with how dyads approach research‐related decisions. Further, for individuals who are determined to have marginal capacity in STEP 6, having a reliable and trusted other present can facilitate supported decision making, a process in which they receive assistance from someone to make their own decision [7]. For example, this trusted other might agree to augment the prospective participant's cognitive functions—by taking notes or helping to weigh pros and cons—as they reach a decision about research participation.

### 2.6 STEP 6: Specify the Process for Assessing Prospective Participants' Decisional Capacity

While it is beyond the scope of this paper to provide a comprehensive review of existing tools, well‐validated, practical means for researchers to assess decisional capacity exist and continue to be developed [18, 19]. Researchers can choose the tool that makes sense in the context of their research. For example, the MacArthur Competence Assessment Tool for Clinical Research (MacCAT‐CR), a semi‐structured interview that evaluates all four decisional abilities, is frequently used and has been validated in older adults, though it has limitations, including the requirement for a trained administrator and a length of roughly 15 min. Another approach to capacity assessment is conversation‐based [20]. The researcher should pre‐specify important points about the research and have set questions for assessment (e.g., “Is what we're talking about research or clinical care?” or “How would participation in this study affect you?”); pre‐specification should limit inter‐rater variability.

Thought should also be given to whether capacity can be assessed in ways that better support people with cognitive impairment and give them the best chance of making decisions for themselves. For example, a researcher enrolling people living with dementia may try to avoid late afternoons or evenings, when neuropsychiatric symptoms can worsen. Researchers may wonder if a formal capacity assessment is always necessary. In some instances, a prospective participant's diagnosis or clinical situation may be highly suggestive of incapacity, and informal screening drawing on researcher experience may quickly confirm this to be true. Say, for instance, the individual is sedated and on a ventilator. Still, there is a dignitary benefit to seeing the individual before deciding they lack capacity.

Researchers should not outsource capacity assessment to individuals not on the research team. Conversations with a family member or a clinician about a prospective participant's decisional abilities, such as the ability to attend to a conversation, may be informative, but it is inappropriate to ask such individuals whether the prospective participant has or lacks capacity. Whether a person has or lacks capacity is answered by an assessment of capacity by the researcher or their designee [11].

### 2.7 STEP 7: Specify How Surrogates Will Be Identified and Contacted When Prospective Participants Are Determined to Lack Decisional Capacity

Individuals determined to have decisional capacity will be able to make their own decisions about research participation. For individuals determined to lack capacity, it will be necessary to identify a surrogate or legally authorized representative who can give consent to research participation on the individual's behalf.

The surrogate may be someone designated by the individual; this may have happened previously or—because the threshold for choosing a surrogate is often lower than the threshold for consenting to research participation—the individual may have the capacity to designate a surrogate. Alternatively, the surrogate may be identified following relevant local laws or policies. For example, state laws regarding surrogate decision makers for clinical care are often relevant to research. There is documented variation in how IRBs handle surrogate consent for adults lacking decisional capacity [21]. We suggest that researchers proactively communicate with their IRB.

Depending on the study's sampling strategy, there may be barriers to identifying and contacting a surrogate. Researchers seeking to recruit participants they have identified via the electronic medical record—for instance, using a diagnostic or billing code—often find that surrogates' names are not clearly recorded or easily discoverable in the electronic medical record. If the researcher sends a recruitment letter to a prospective participant at their home address or messages them via an online patient portal, the surrogate may or may not see the study‐related information, which can lead to accrual problems. If the researcher reaches out to a surrogate directly, there may be concerns about sharing the prospective participant's protected health information that implicate privacy laws; the IRB can be a resource in understanding or addressing such concerns.

Once a surrogate is identified and contacted, the surrogate should be given the same information that would be given to any prospective participant (STEP 4). Box 3 includes information about how surrogates make research‐related decisions.

BOX 3 **How surrogates make research‐related decisions.**

Ideally, a surrogate will use information about the study to approximate the decision the prospective participant would have made for themselves if able; this is known as a “substituted judgment.” A surrogate may rely on a prospective participant's express wishes or reason from analogous prior circumstances when making their decision. Though there has been discussion of specific research advance directives—that is, a document communicating an individual's preferences regarding research participation—in the literature, uptake remains limited in practice, and in the United States, they are not legally binding [22, 23, 24]. The substituted judgment standard is meant to show respect for autonomy (i.e., self‐determination).

Studies of surrogate decision making have repeatedly found that what individuals say they would want and what their surrogates think they would want do not always match [25]. Given that substituted judgment is grounded in respect for autonomy, this mismatch is concerning. Just how concerning depends on how strictly people want their preferences followed [26]. It is, perhaps, reassuring that prior studies suggest older Americans broadly support surrogate decision making, and a majority would grant their surrogate some or complete leeway when making decisions [27].

If a surrogate lacks knowledge of the prospective participant's research‐related preferences and cannot reason from analogous prior circumstances, the surrogate's decision should be based on a “best interests” standard. This is necessary when the prospective participant has never had decisional capacity (e.g., due to intellectual or developmental disabilities). The best interests standard, which focuses on well‐being, is grounded in considerations of beneficence.

Surrogate decision making can be a difficult task. The perceived burden is influenced by various factors, including the risk and nature of the study (i.e., greater risk, greater burden), the extent to which the prospective participant is able to contribute to decision‐making (i.e., greater ability, less burden), and the surrogate's prior experiences making decisions on the individual's behalf (i.e., greater experience, less burden) [28]. Researchers would do well to acknowledge this burden when talking with surrogates.

Note that needing a surrogate to give consent on a prospective participant's behalf may make research participation relatively more difficult (or even impossible) for unrepresented individuals, a population no less in need of evidence‐based interventions to promote their health and well‐being.

### 2.8 STEP 8: Determine Whether There Are Ways to Involve Prospective Participants Who Lack Decisional Capacity in the Decision‐Making Process

A person found to lack capacity may nevertheless have some of the underlying decisional abilities. For example, they may comprehend that they are being asked to participate in research focused on dementia and communicate a desire to enroll, but they cannot comprehend important study risks. Given the ethical requirement to allow people, to the extent that they are capable, to make decisions about what will or will not happen to them, the strong default should be incorporation of strategies short of consent to demonstrate respect for prospective participants who lack capacity but maintain some decisional abilities.

Two common strategies researchrs use are to seek a prospective participant's assent or to provide the prospective participant an opportunity to dissent. Assent is an affirmative agreement to participate in research. To be capable of assenting to research participation, the prospective participant should have at least minimal comprehension of some aspects of study participation and be able to communicate a choice [29]. Assent serves many of the functions of consent [30], but it is insufficient on its own to authorize enrollment in a study. Thus, a surrogate must still grant consent. The absence of a regulatory requirement that assent be obtained from adults who lack decisional capacity has been a source of concern for ethicists [28].

Dissent, whether verbal or non‐verbal, is an objection to research participation [7]. Typically, it does not require a reason or justification, and the individual dissenting generally does not have to demonstrate any comprehension for their dissent to be respected. A notable challenge is that it may be difficult to discern whether a behavior, such as walking out of an exam room, is an indication of dissent or rather a symptom or condition, such as agitation, that needs to be treated. In light of this challenge, researchers can attempt to address prospective participants' initial distress or concerns. Input from a surrogate, caregiver, or other informant may be useful to understand possible causes and potential solutions. Flexibility to address distress or concerns is not license to cajole the prospective participant. From an ethical perspective, a prospective participant's dissent should typically be determinative if—after a reasonable effort to address any distress or concerns—it is sustained; this holds even if the prospective participant's surrogate is willing to grant consent over the prospective participant's objections [29]. In narrow circumstances, exceptions are possible, such as if participation in research offers a prospect of direct benefit that is only available through research participation.

Operationalizing assent and dissent depends on context; researchers should, therefore, prospectively detail what standards and processes they will use. What remains consistent is that if an individual who has the ability to assent to research participation does not, if an individual makes a sustained and clear objection to research participation, or if an individual's response remains ambiguous, they should typically not be enrolled in research [29].

### 2.9 STEP 9: In Longitudinal Studies, Consider How Capacity and Consent Might Change Over Time

In longitudinal studies where deterioration of participant capacity is expected—for example, an observational cohort study of people at risk for dementia—scheduled reassessments of capacity are not necessary. Instead, researchers should adopt a functional approach. This entails an ongoing observation of participants' ability and willingness to participate in the research. Changes, such as difficulty scheduling or completing research‐related procedures, warrant a capacity assessment. If a participant has lost capacity, the appropriate surrogate should be asked for consent; ideally, this surrogate was identified at the time of enrollment in the event they would be needed later, and preferences regarding ongoing research participation were discussed.

## 3 Conclusion

Research ethics guidelines and regulations governing human subjects research, including the Common Rule, emphasize the importance of consent for research participation. The consent process affords individuals an opportunity to make informed, voluntary decisions that participation is (or is not) right for them. Yet some adults lack decisional capacity and are considered vulnerable becasue they cannot give informed consent for their own research participation. It is essential to conduct research to advance evidence‐based care for these populations. As a result, those lacking capacity cannot be categorically excluded from research but instead merit additional or special protections when their enrollment is justified. The nine‐step framework outlined herein offers a way of systematically thinking through relevant ethical and practical issues.

## Author Contributions

All authors meet the criteria for authorship stated in the Uniform Requirements for Manuscripts Submitted to Biomedical Journals. E.A.L. made substantial contributions to the conception of the work and drafting the work. J.K. and S.M. reviewed the work critically for important intellectual content. All authors approved the final version for publication.

## Funding

This work was supported by the National Institute of Aging (NIA) of the National Institutes of Health under Award Number U54AG063546, which funds NIA Imbedded Pragmatic Alzheimer's Disease and AD‐Related Dementias Clinical Trials Collaboratory (NIA IMPACT Collaboratory). Drs. Largent and Karlawish are supported by R01AG077111. The content is solely the responsibility of the authors and does not necessarily represent the official views of the National Institutes of Health.

## Disclosure

The sponsor played no role in the design, methods, subject recruitment, data collection, analysis, and preparation of the paper.

## Conflicts of Interest

The authors declare no conflicts of interest.
